# Validating the Health Literacy Promotion Practices Assessment Instrument

**DOI:** 10.3928/24748307-20171030-01

**Published:** 2017-12-11

**Authors:** Allison P. Squires, H. Shonna Yin, Simon A. Jones, Sherry A. Greenberg, Ronnie Moore, Tara A. Cortes

## Abstract

**Background:**

How health care professionals address health literacy as part of the provider-client relationship is important for prevention and promoting self-management and symptom management. Research usually focuses on patients' health literacy and fails to examine provider practices, thus leaving a gap in the literature and patient outcomes analyses.

**Objective:**

The study tested the reliability and validity of a series of questions developed to evaluate health care provider health literacy promotion practices on an interprofessional sample.

**Methods:**

This exploratory cross-sectional study took place between 2013 and 2015. Participants included graduate level health professions students from nursing, midwifery, medicine, pharmacy, and social work. Exploratory factor analyses with varimax rotation examined the reliability and validity of the instrument as a measure of health literacy promotion practices.

**Key Results:**

Of the participants in the programs, 198 completed the health literacy questions in the online survey. Exploratory factor analysis showed that questions loaded on two factors connected with either individual or organizational characteristics that facilitated health literacy promotion practices. The Cronbach's alpha for the instrument was 0.95.

**Conclusions:**

This study helped determine the reliability and validity of the items as measures of providers' health literacy practices. Future research will help to further establish the stability of the instrument as a measure and increase its potential reliability when linking provider practices to health literacy sensitive client outcomes. Testing the instrument separately and concurrently with each health profession is recommended until instrument stability across professional roles has been established. **[*Health Literacy Research and Practice*. 2017;1(4):e239–e246.]**

**Plain Language Summary:**

We sought to develop a survey instrument people could use to assess how health care providers help patients understand their health better. After getting responses from 198 health care providers, we ran statistical tests to check the quality of the questions for measuring provider practices. We found the questions were good at evaluating provider practices around promoting patient understanding of health issues.

Health care professionals have a collective responsibility to deliver care to clients in a meaningful, understandable way in all health care encounters. Addressing health literacy is an essential health promotion strategy for improving individual and population health as well as for reducing health disparities (Parker, 2000; Peerson & Saunders, 2009; Ratzan, 2001). Some actions by providers may help address a client's health literacy deficits, whereas others may help them overcome barriers to health literacy, a far more complex problem because these may include social, economic, and contextually driven barriers that are beyond the control of the provider.

Health literacy promotion in clinical practice is a function of the concept of intersectionality—where all that comprises a person's identity influences his or her interactions with other people and the world around them, often simultaneously (DeFrancisco, Palczewski, & McGeough, 2014). The dynamics between clients and providers during a health care encounter result from the intersectionality of each other's identities and may affect health literacy. For example, a provider with poor health literacy promotion practices will likely find that outcomes are not falling within expected normal ranges. Although other client factors will contribute to the individual's overall health literacy (Beauchamp et al., 2015; Lee, 2006; Martin et al., 2009), capturing the extent of the provider's contribution to health literacy associated outcomes is an important step toward addressing overall deficiencies in health literacy.

Most studies that examine the issue of health literacy focus on patients. Few studies have examined how to measure health care provider health literacy promotion practices with patients (Altin, Finke, Kautz-Freimuth, & Stock, 2014). In 2007, drawing from a series of actions identified by the American Medical Association to promote health literacy, Schwartzberg, Cowett, Vangeest, & Wolf (2007) identified 14 techniques that are most commonly used by providers to promote health literacy in their clients. They assessed the frequency of these practices among conference attendees who self-identified as physicians, registered nurses, or pharmacists. Another study by Turner et al. (2009) tested similar questions on a group of pediatricians, but identified three additional strategies that providers commonly used. A limitation of both studies is that neither evaluated the reliability and validity of the questions as measures. Because health literacy promotion is a collective responsibility of all members of the health care team, a reliable and valid instrument that is stable across health professions would be a welcome resource. Therefore, through three interprofessional health workforce capacity building projects, our team of researchers sought to test the reliability and validity of the 17 items identified by Schwartzberg et al. (2007) and Turner et al. (2009) as “most frequent practices” on an interprofessional sample of registered nurses, nurse practitioners, midwives, physicians, social workers, and pharmacists.

## Methods

This cross-sectional pilot study occurred between 2013 and 2015. The 17 health literacy promotion practices items previously tested but not validated in the literature served as the instrument's foundation. These items were part of a larger pre-intervention survey used in the programs that included questions about interprofessional team work and a demographic profile. The health literacy questions were the second section in the large survey, after the interprofessional team work instrument. Institutional Review Board approval was received from New York University.

## Study Participants

Study participants were eligible if they were graduate students in nursing, midwifery, pharmacy, social work, or medicine (residents), as well as if they were a part of 1 of 3 Health Resources and Services Administration-funded programs taking place in a large urban private university. Each program involved an interprofessional educational intervention designed to build capacity in geriatric care delivery and included health literacy promotion as part of the intervention.

Because a primary goal of this analysis was to assess the reliability and validity of the items as a single instrument, the target sample size needed was a minimum of three respondents (10 being the ideal number) per item (DeVellis, 2017; Nunnally & Bernstein, 1994). With an instrument comprised of 17 questions, the sample size needed was between 51 and 170 participants.

## Pre-Data Collection Content Validation

Content validation of items is necessary to assess prior to data collection to improve the reliability and validity of an instrument overall (DeVellis, 2017). The content of the survey items should be linked to the concepts being studied and how they are constructed in reality (DeVellis, 2017). This step is integral to enhancing the overall reliability and validity of survey instruments.

Because content validation had only occurred with physician groups in the previous studies, prior to administering the survey the team used an interprofessional team of health care professionals: eight physicians (one pediatrician, six primary care, one emergency medicine), two health care social science researchers with careers built on studying medical education and physician practice, five registered nurses, two nurse practitioners (adult-geriatric primary care), two social workers, and one pharmacist to review the instrument for face validity and participate in a content validation exercise. The technique used for content validation was content validity indexing (CVI). CVI is a quantifiable approach that uses 5 to 10 expert raters to evaluate the relevance of survey items for their use with the intended audience (Polit & Beck, 2006; Polit, Beck, & Owen, 2007). The analysis produces both item level and scale level scores, with an item level score (I-CVI) that should rank at 0.70 or higher for inclusion and a scale level score (S-CVI) of 0.80 for content validity to be present (Polit et al., 2007). When using a modified kappa score calculation to correct the CVI score for chance agreement by raters, the scale shifts to >0.74 = excellent, 0.60–0.73 = good, 0.5–0.59 = fair, and <0.50 = poor (Ciccheti & Sparrow, 1981). The technique is also useful for identifying potentially problematic items that may be at higher risk for missing data and items that may be sensitive to participant identity (Squires et al., 2013).

When implementing the CVI exercise, the physicians on the team were concerned that their perspectives on health literacy promotion practices would vary from other health care professionals because of professional socialization. To address this concern, the CVI exercise was conducted by one team of physicians that included the two social science researchers who had worked in medical education and physician practice analyses studies and one interprofessional team comprised of nurses, social workers, and pharmacists. The exercise was also completed to assess the hypothesis that professional socialization would produce varying perceptions of “relevance” of certain health literacy promotion practices. To complete the exercise, each team rated the items on a scale of 1 to 4 with 1 = *not relevant* and 4 = *highly relevant*.

Data analysis of the raters' scores was conducted using the formula provided by Polit et al. (2007), which corrects for chance agreement among the raters by generating a modified kappa score. Research suggests that the modified kappa score offers more flexibility with regard to item selection when compared to the CVI score alone; items receiving a kappa score of 0.60 or higher (rated “good” on this scaling measure with >0.74 as excellent) are considered acceptable indicators of content validity (Squires et al., 2013).

## Data Collection

Once content validation was completed, seven separate survey periods were used for data collection over the course of the 3 years that the programs operated. All participants were emailed the survey via the Qualtrics (Provo, Utah)survey management system timed prior to the start of the interprofessional education intervention at periods deemed appropriate by their program chairs. Each participant had at least three reminders sent. Survey periods lasted 3 weeks and were extended as necessary until at least a 50% response rate was achieved, a level considered acceptable for Internet-based surveys (Johanson & Brooks, 2010; Michael Bowling et al., 2006). Responses were tied to participant email addresses, which were removed prior to data analysis to ensure respondent confidentiality. Respondent IP addresses were also collected to ensure unique responses and removed prior to data analysis.

## Data Analysis

Upon completion of data collection, data were cleaned and de-identified for analysis in 2016. Incomplete surveys were eliminated from the final sample used for analyses. Using R statistical software package (Version 3.2.2), the team conducted exploratory factor analyses (EFA) as the first step in validating the items as a collective measure of health literacy promotion practices. The EFA incorporated varimax rotation to facilitate interpretation and achieve simple structure of the instrument. It is also a step that would moderate any effects of professional sample size differences (DeVellis, 2017). Eigenvalue cut offs were set at 1 for factor extraction. Factor solutions were examined at 0.4, 0.5, and 0.6 factor loading cut off levels. Cronbach's alpha was calculated to evaluate the reliability of the instrument.

## Results

The results divide into two sections—content validation and factor analysis—that outline the results of the reliability and validity assessment of the instruments. The results are based on a final sample size of 198 participants who had answered all of the health literacy practices questions. The sample size was more than sufficient to conduct the factor analyses and assess overall reliability and validity of the questions as measures of health literacy promotion practices. Demographically, participants had an average age of 28 years, 67% were women, and had an average of 5.5 years of work experience (range, 1 to 21 years) with an average of 4 years of work experience in health care (range, 1 to 18 years). Consistent with most graduate level health professions education programs, most respondents (67%) were currently employed and working in health care in some capacity.

By profession, survey participants included 22 nurse practitioner and nurse midwifery students, 19 social work students, 19 resident physicians, and 138 pharmacy students. As the aim of this study was to validate the instrument and examine the factor structure, not to compare performance between groups or by role, the distribution of the sample was not a concern for the team because psychometric analyses emphasize evaluating item performance. Additionally, no research has shown that provider role influences factor analysis results.

## Content Validation Results

The CVI exercise demonstrated that some item-level variations in perceived relevance of items occurred between professions, with these results shown in **Table 1**. Physician raters differed in their item relevance ratings from the other health care professionals on 7 of 17 items. Only on 1 item (HL 12: “Follow up phone calls”) did the physician raters rate relevance higher than the other health care professionals. Five items (HL 10, 12, 13, 14, 16) were identified through their modified kappa scores of ≤0.60 as potentially problematic by physicians. The second group of raters identified three items as potentially problematic (HL 10, 12, 14), matching with the physician group on HL 12 and 14. Ultimately, all items were perceived as broadly relevant to health literacy practices across all health professions, as indicated by the respective S-CVI scores by the two groups: physician group with 0.74 modified kappa score (excellent) and the interprofessional group with a 0.87 modified kappa score (excellent).

## Factor Analysis Results

**Figure 1.**
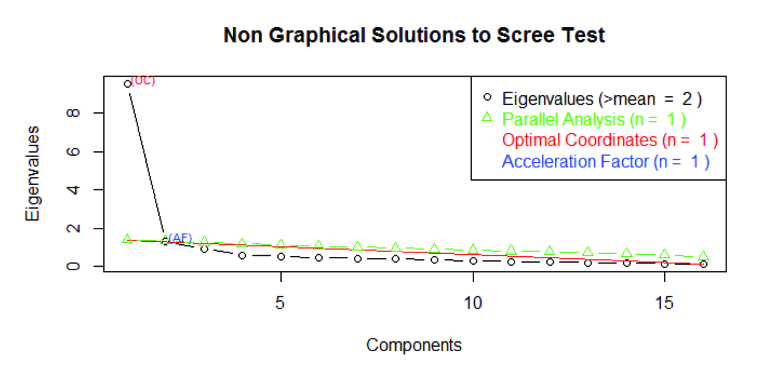
Scree plot results from the exploratory factor analysis.

For the factor analysis, **Figure 1** illustrates the results of the scree plot. The Eigen value analysis suggests a two-factor solution. Parallel analysis, optimal coordinates, and acceleration factors suggest a one-factor solution. A one-factor solution, however, explained only 57% of the variance that was consistent at all factor cut off levels, including at 0.6.

For the two-factor solution with varimax rotation, cutoffs were again repeated at 0.4, 0.5, and 0.6. At 0.4 in the two-factor solution, half the items loaded on both factors. At 0.6, 5 items did not load on either factor. Therefore, the 0.5 cutoff presented the best factor solution with all items loading on at least one factor and explained 63% of the variance. **Table 2** shows the factor loadings and provides the instrument's items. Finally, the Cronbach's alpha score for the instrument was 0.95, suggesting excellent reliability and strong internal consistency.

## Discussion

The results of the instrument validation process suggest that the 17 items previously tested by Schwartzberg et al. (2007) and Turner et al. (2009) can collectively serve as a reliable and valid measure of health care providers health literacy promotion practices. The instrument has the potential to be used as a practice measure where results could be linked to client outcomes in future research studies.

We suggest calling the questions the “Health Literacy Promotion Practices Assessment” instrument. With further testing, the two factors may solidify into two subscales that we suggest naming “Organizational Resource Influenced Practices” (Factor Set 1) and “Individually Influenced Practices” (Factor Set 2).

Few studies have addressed the role of the organization in health literacy promotion practices, although increased attention to the issue is growing as professionals increasingly practice within organizations and less in private practice (Brach et al., 2012; Kowalski et al., 2015). Organizational support is not only having educational resources available to providers to use to teach their patients, but also that management practices support health care professionals being able to take the time to address health literacy with their clients. The results of the factor analysis support the evidence that emphasizes the importance of the organization in supporting health literacy promotion practices. Interestingly, none of the items in the instrument directly address organizational resources for health literacy promotion.

Combining the current instrument with one that can more precisely discern the effect that different organizational cultures have on health literacy practices would also be a welcome contribution to the literature. Work on a health literate organizational assessment instrument (Kowalski et al., 2015), which is based on characteristics of health literate organizations (Brach et al., 2012), is already in progress. Combining both instruments in a single study or training intervention would create a powerful tool that could help establish the links between organizational resources and culture and health care professional's health literacy practices with their clients.

## Limitations

Limitations of the study mostly relate to sample composition. To achieve an adequate pharmacy sample, we had to survey all pharmacy students (all of whom were eligible to participate in the interprofessional collaboration intervention through clinical placement processes), which led to the sample imbalance by profession. However, large sample sizes are important for improving the overall stability of factor analysis results (DeVellis 2017). Including the full sample of the pharmacy students allowed us to achieve more reliable factor analysis results than if we had limited the sample to only nurses, midwives, physicians, and social workers (*n* = 60). A factor analysis conducted only on that sample would have met the minimum three participants per item ratio, but would have produced less stable results. And as stated previously in the methods, there is no evidence to suggest that dominance of one provider in a sample size would influence factor analysis results. Certainly, the predictive validity of the instrument on provider practice patterns would be influenced if we were comparing survey results by professional group, but that was not the intent of this study.

## Future Research

Although the Health Literacy Promotion Practices Assessment demonstrates potential as a reliable and valid measure of health care professions health literacy practice, more research is needed to confirm its reliability as a measure. This article was a first step. A larger cross-sectional study with a balanced sample by profession, which should also include physical and occupational therapists, would help to further confirm its reliability and validity as a measure and help determine if and where health literacy promotion practice variation occurs between professionals. Sample sizes of at least 100 providers per role with factor analyses conducted on each role would help establish the extent of variation, if any, in the instrument's stability in measuring practice variations by provider role. This would help to further establish the stability of the instrument across health care professions and facilitate its use in interprofessional interventions aimed at improving provider health literacy practices with patients.

## Conclusions

This study determined that the 17 items can be collectively considered as reliable and valid measures of health literacy promotion practices by health care professionals. Determining the practices providers use to address the health literacy of their clients during health care encounters is an important part of improving an individual's health literacy. As we attempt to develop and refine measures of our practices and link them to health literacy sensitive outcomes, we can further enhance clinical practice in this area, solidify practice competencies, and facilitate interprofessional team-based approaches to health literacy promotion.

## Figures and Tables

**Table 1:** Content Validity Indexing Results

**Review Group and Number**	**Item Score Type**	**HL 1**	**HL 2**	**HL 3**	**HL 4**	**HL 5**	**HL 6**	**HL 7**	**HL 8**	**HL 9**	**HL 10**	**HL 11**	**HL 12**	**HL 13**	**HL 14**	**HL 15**	**HL 16**	**HL 17**	**Scale CVI**
MD/SSR	I-CVI	1	**0.70**	**0.70**	0.90	0.80	1	0.90	**0.70**	1	**0.50**	0.80	**0.60**	**0.30**	**0.30**	1	**0.40**	0.90	0.74^a^
*n* = 10	*k*	1	0.70	0.70	0.90	0.80	1	0.90	0.70	1	**0.50**	0.80	0.60	**0.29**	**0.29**	1	**0.39**	0.90	0.73^a^
RN/SW/PH	I-CVI	1	1	0.90	1	0.80	1	1	1	1	0.80	**0.50**	**0.50**	0.80	**0.50**	1	1	1	0.87^b^
*n* = 10	*k*	1	1	0.90	1	0.80	1	1	1	1	0.80	**0.50**	**0.50**	0.80	**0.50**	1	1	1	0.87^b^

Note. Bold indicates items that received “low relevance” scores. HL= health literacy; I-CVI = item-content validity indexing; *k* = kappa score; MD = physician, PH = pharmacist; RN = registered nurse or nurse practitioner; SSR = social science researcher; SW = social worker.

a Physician/SSR scale-CVI.

b Nursing/social work/pharmacy scale-CVI.

**Table 2:** Factor Analysis Results

**Item**	**Statement**	**Factor 1: Organizational Resource-Influenced Practices**	**Factor 2: Individually Influenced Practices**

1	Use simple language (avoid technical jargon)	-	-

2	Hand out printed materials to clients	-	0.61

3	Speak more slowly	-	0.76

4	Read aloud instructions	-	0.77

5	Write out instructions	0.57^a^	0.55

6	Present two or three concepts at a time and check for understanding	0.53	0.63^a^

7	Ask clients how they will follow instructions at home	-	0.66

8	Ask clients if they would like a family member involved in the discussion	-	0.56

9	Ask clients to repeat information, use a Teach-Back technique	0.50	0.58^a^

10	Have client follow up with office staff to review instructions	0.74	-

11	Draw pictures	0.80	-

12	Follow up with telephone call to check understanding/compliance	0.64	-

13	Use models to explain	0.87	-

14	Select educational materials that are written at a literacy level appropriate for clients	0.58^a^	0.56

15	Develop educational materials that are written at a literacy level appropriate for clients	0.77	-

16	Identify that a client has a literacy problem	0.51	-

17	Underline key points in client information handouts	0.50	0.59^a^
	SS loadings	5.19	4.96
	Proportion variance	0.32	0.31
	Cumulative variance	0.32	0.63

Note.

a Highest factor loading score. SS = sum of square.
